# SAMDC Activity Is Involved in the Melatonin-Mediated Tolerance of Barley (*Hordeum vulgare* L.) Seedlings to Osmotic Stress

**DOI:** 10.3390/plants15203114

**Published:** 2026-10-12

**Authors:** Yunxia Chang, Jinming Yu, Congmin Hua, Huaipan Liu

**Affiliations:** 1 College of Life Science and Agronomy, Zhoukou Normal University, Zhoukou 466001, China; changyx2006@126.com; 2 State Key Laboratory of Cotton Biology, Henan University, Kaifeng 475000, China

**Keywords:** barley (*Hordeum vulgare* L.), melatonin, enzyme activity, anti-oxidation, compatible solutes, photosynthetic characteristics

## Abstract

Exogenous melatonin can elevate the tolerance of plants to drought stress. The mechanism of melatonin-mediated tolerance has been extensively studied. Numerous studies have shown that melatonin treatment can increase contents of osmotic adjustment substances, alleviate oxidative damage, and maintain photosynthetic characteristics of plants under drought stress. However, a research gap remains, i.e., whether melatonin plays the above-mentioned functions by regulating polyamine metabolism. In the present study, *S*-adenosylmethionine decarboxylase (SAMDC) activity and the levels of spermine and spermidine were detected in melatonin-treated barley (*Hordeum vulgare* L.) seedlings under polyethylene glycol-6000, which was used to simulate drought stress. The aim was to probe whether polyamine metabolism was involved in the process of melatonin-mediated osmotic stress tolerance. The results indicated that exogenous melatonin improved the tolerance of barley seedlings to osmotic stress, coupled with increasing content of proline, alleviating oxidative damage and maintaining the photosynthetic characteristics of barley seedlings. More importantly, melatonin elevated the activity of SAMDC, and thereby the contents of spermine and spermidine. Thus, it was speculated that melatonin functioned by regulating polyamine metabolism. The hypothesis was further reinforced by the results of inhibitor experiments. Inhibitor methylglyoxal-bis (guanylhydrazone), which can inhibit SAMDC activity strongly and specifically, not only inhibited the melatonin-induced increases in the activity of SAMDC and the levels of spermine and spermidine, but also inhibited melatonin-induced increases in antioxidant ability, proline content, and photosynthetic capacity. The relationship between SAMDC activity and the effects of melatonin on proline content, antioxidant ability, and leaf photosynthetic characteristics was further explored and is discussed below. Therefore, it could be concluded that SAMDC might be involved in the melatonin-mediated tolerance of barley (*Hordeum vulgare* L.) seedlings to osmotic stress.

## 1. Introduction

Plant growth, development, and yield are severely affected by environmental stresses, such as drought, high temperature, heavy metals, and salt stresses [1,2,3]. Due to climate change and the increasing shortage of water resources, drought stress has become an issue of global focus [4]. It directly affects the physiological and biochemical processes of plants, such as stomatal conductance, cell dehydration, and membrane lipid peroxidation, as well as the decreases in antioxidant and leaf photosynthetic capacities, and thereby affects plant growth, development, yield, and even survival [5,6]. Barley (*Hordeum vulgare*), which is often used to make beer, is widely cultivated all over the world. Drought presents a significant limitation to barley yield in arid and semi-arid areas worldwide. Therefore, it is particularly important to enhance the tolerance of barley to drought stress [7]. Extensive studies have been conducted to enhance the drought tolerance of crops and to mitigate injury in crops. Exogenous plant growth substances play a pivotal role in adjusting plant growth and development, as well as enhancing their stress tolerance. For example, exogenous melatonin application is increasingly investigated.

It is well known that N-acetyl-5-methoxytryptamine (melatonin) is a multifunctional molecule that plays a crucial role in plant growth, development, and stress response [8,9,10,11,12,13,14], especially in plant drought stress response [9,10,11,12,13]. Moreover, increasing attention has been paid to research on the mechanism of melatonin-mediated drought resistance [15,16,17,18]. Drought negatively impacts crop growth and yield, but variations in melatonin-treated crops and their mechanisms of adaptation determine their sensitivity to drought and their ability to withstand adverse conditions. For example, Noor and Sheng [13] have reported that exogenous melatonin can enhance photosynthetic performance, improve antioxidant capacity, and thereby alleviate drought injury in maize (*Zea mays* L.). A study by Zhang et al. [11] has showed that exogenous melatonin can maintain photosynthetic organs, decrease reactive oxygen species level, and thereby elevate the tolerance of licorice (*Glycyrrhiza uralensis* Fisch.) seedlings to drought stress. A review by Dzinyela et al. [10] elucidates that melatonin-mediated phytohormonal crosstalk plays crucial roles in improving plant tolerance against drought stress. Melatonin may exert its effects through signal transduction. The binding of melatonin and the receptor in the bio-membrane activates downstream heterotrimeric G proteins, which trigger rapid intracellular signaling cascades. These cascades include activating the MAPK pathway and modulating ion channel activity, resulting in swiftly regulating stomatal closure in response to physiological processes such as drought stress. Additionally, melatonin has been demonstrated to regulate the plant stress response by directly inhibiting the accumulation of reactive oxygen species [17]. Overall, these studies have revealed that melatonin application can elevate plant drought tolerance by improving antioxidant and photosynthetic capacity and increasing the contents of osmotic adjustment substances. However, polyamine metabolism, especially SAMDC activity, is scarcely mentioned in studies on the mechanism of melatonin-mediated drought tolerance.

Polyamines, including mainly putrescine, spermidine, and spermine, are pleiotropic bioorganic plant regulators and are involved in plant growth and development, as well as adaptation to diverse abiotic and biotic stresses [19,20,21,22,23]. Putrescine is mainly synthesized by the decarboxylation reaction of ornithine and arginine, catalyzed by ornithine decarboxylase and arginine decarboxylase, respectively [24,25]. Through the catalysis of spermidine synthase, one aminopropyl residue is connected to one putrescine chain end to form spermidine. By the catalysis of spermidine synthase, another aminopropyl residue is connected to the other putrescine chain end to form spermine. *S*-adenosyl-L-Met is the important precursor for the biosynthesis of spermidine and spermine. Although spermine and spermidine synthases play roles in spermine and spermidine biosynthesis, *S*-adenosyl-L-methionine decarboxylase (SAMDC) is the key enzyme in the biosynthesis reactions [26,27]. Studies have shown that spermine and spermidine enhance plants’ resistance to environmental stress by directly eliminating reactive oxygen species [22,28,29,30].

Polyethylene glycol 6000 (PEG-6000) is widely used as an osmotic regulator to induce osmotic stress by simulating water potential. Therefore, in this experiment, melatonin was selected to be supplied to the barley seedlings under PEG osmotic stress. In this study, to elucidate whether polyamine metabolism is involved in the melatonin-mediated tolerance of barley seedlings to osmotic stress, two intuitional indexes, root length and relative increasing rate of barley seedling dry weight (RIRBSDW), which could indicate seedling growth, were first measured, followed by determinations of SAMDC activity and the levels of polyamines. Then, to further evaluate the changes in seedling tolerance, osmotic adjustment capacity (indicated by detecting the levels of proline and soluble proteins) and antioxidant abilities (shown by evaluating oxidative damage and eliminating reactive oxygen species) were detected. Finally, leaf photosynthetic performance, which could finally reflect the degree of seedling tolerance, was also evaluated.

It was found that exogenous melatonin could improve the tolerance of barley seedlings to osmotic stress. Furthermore, SAMDC activity and the contents of spermine and spermidine increased significantly. Concurrently, antioxidant capacity, osmolyte contents, and photosynthetic performance were also improved. Therefore, it could be speculated that melatonin functioned by elevating the activity of SAMDC and thereby the contents of spermine and spermidine. The hypothesis was further reinforced by the experimental results with methylglyoxal-bis (guanylhydrazone) (MGBG) inhibitor, which can strongly and specifically inhibit SAMDC activity [31].

## 2. Results

### 2.1. Melatonin Enhances Seedling Growth Under Osmotic Stress

**Figure 1:**
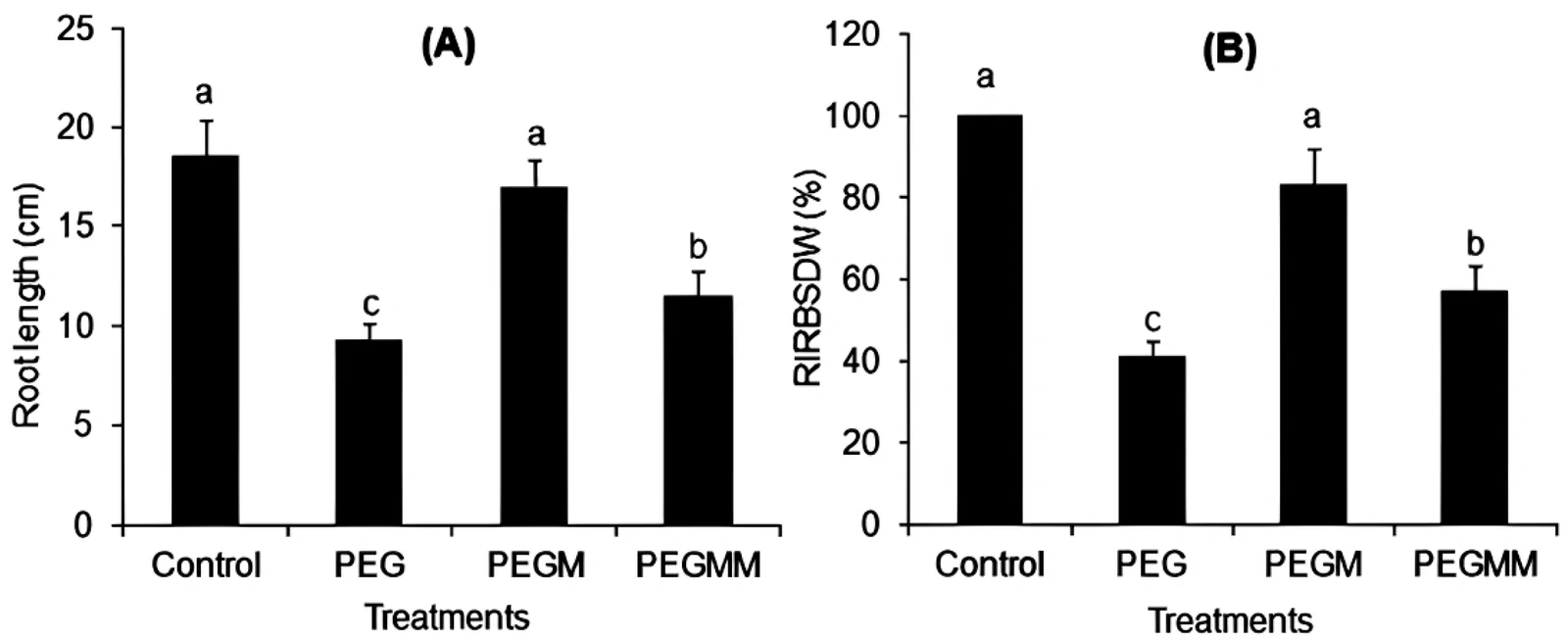
Melatonin enhances seedling growth under osmotic stress ((**A**): root length; (**B**): RIRBSDW: relative increasing rate of barley seedling dry weight). Seedlings were cultured in full-strength (−0.15 MPa) Hoagland solution and marked as “Control”. Seedlings were cultured in full-strength Hoagland solution with PEG: polyethylene glycol (−0.85 MPa) (marked as “PEG”), PEG (−0.85 MPa) + melatonin (0.1 mM) (marked as “PEGM”), and PEG (−0.85 MPa) + melatonin (0.1 mM) + MGBG: methylglyoxyl-bis (guanylhydrazone) (0.5 mM) (marked as “PEGMM”), respectively. The data in the figure are the averages of nine independent tests ± standard error. Two-way analysis of variance (ANOVA) was used for significant-difference analysis among treatments, followed by Tukey’s Honest Significant Difference (HSD) test. The values associated with the different lowercase letters over the column were significantly different according to HSD at *p* ≤ 0.05, while data of each column indicated by the same letters are not significantly different following Tukey’s HSD test (*p* > 0.05).

Two typical parameters, root length and RIRBSDW, were used here to evaluate barley seedling growth. From Figure 1, it can be seen that under osmotic stress, root length (Figure 1A) and RIRBSDW (Figure 1B) decreased from 18.5 to 9.3 cm (statistical significance from “a” to “c”) and from 100% to 41% (statistical significance from “a” to “c”), respectively, indicating that PEG could reduce barley seedling growth. Melatonin treatment enhanced the growth of barley seedlings subjected to osmotic stress, because root length and RIRBSDW increased from 9.3 to 16.9 cm (statistical significance from “c” to “a”) and from 41% to 83% (statistical significance from “c” to “a”), respectively. More interestingly, MGBG reversed melatonin effects on barley seedling growth, as root length and RIRBSDW decreased from 16.9 to 11.5 cm (statistical significance from “a” to “b”) and from 83% to 57% (statistical significance from “a” to “b”), respectively. MGBG can inhibit the activity of SAMDC specifically, which plays a key function in the biosynthesis of spermidine and spermine. Hence, it is necessary to analyze polyamine levels and the activity of SAMDC, which are involved in polyamine biosynthesis.

### 2.2. Changes in the Activities of Arginine Decarboxylase and SAMDC in the Leaves of Barley Seedlings Treated with Melatonin

**Figure 2:**
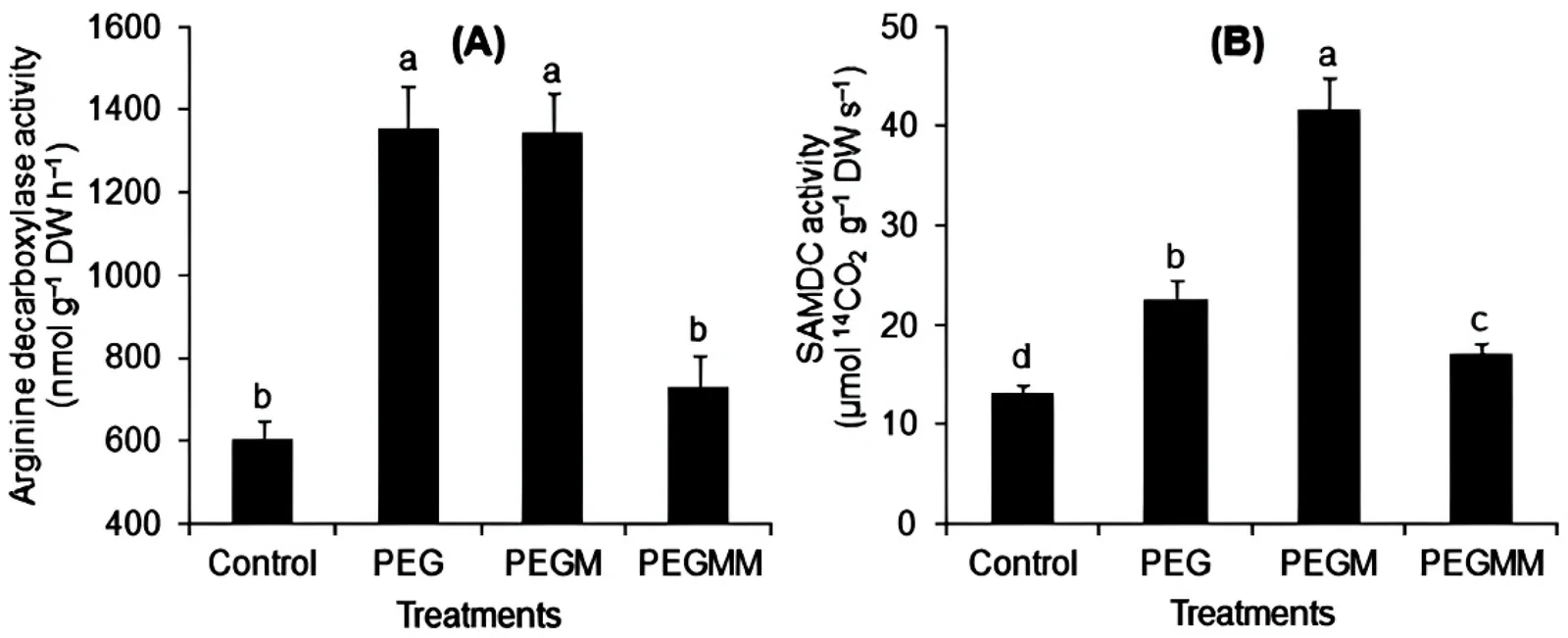
Changes in the activities of arginine decarboxylase (**A**) and SAMDC (**B**) in the leaves of barley seedlings treated with melatonin (DW: dry weight). Seedlings were cultured in full-strength (−0.15 MPa) Hoagland solution and marked as “Control”. Seedlings were cultured in full-strength Hoagland solution with PEG: polyethylene glycol (−0.85 MPa) (marked as “PEG”), PEG (−0.85 MPa) + melatonin (0.1 mM) (marked as “PEGM”), and PEG (−0.85 MPa) + melatonin (0.1 mM) + MGBG: methylglyoxyl-bis (guanylhydrazone) (0.5 mM) (marked as “PEGMM”), respectively. The data in the figure are the averages of nine independent tests ± standard error. Two-way analysis of variance (ANOVA) was used for significant-difference analysis among treatments, followed by Tukey’s Honest Significant Difference (HSD) test. The values associated with the different lowercase letters over the column were significantly different according to HSD at *p* ≤ 0.05, while data of each column indicated by the same letters are not significantly different following Tukey’s HSD test (*p* > 0.05).

Arginine decarboxylase (Figure 2A) and SAMDC activities (Figure 2B) were detected here to further probe the mechanism of the melatonin-mediated tolerance of barley seedlings to osmotic stress. Compared with the control, arginine decarboxylase activity increased (statistical significance from “b” to “a”) in seedling leaves under PEG osmotic stress. The PEG-induced increase in arginine decarboxylase activity was not affected by melatonin treatment (statistical significance from “a” to “a”), but the increase was inhibited by MGBG (statistical significance from “a” to “b”) (Figure 2A). Compared with the control, SAMDC activity in leaves under PEG increased, and it increased nearly two-fold with melatonin plus PEG treatment (statistical significance from “d” to “a”). However, MGBG inhibited the melatonin-induced increase (statistical significance from “a” to “c”) (Figure 2B).

### 2.3. Changes in the Contents of Free Polyamines in the Leaves of Barley Seedlings Treated with Melatonin

**Figure 3:**
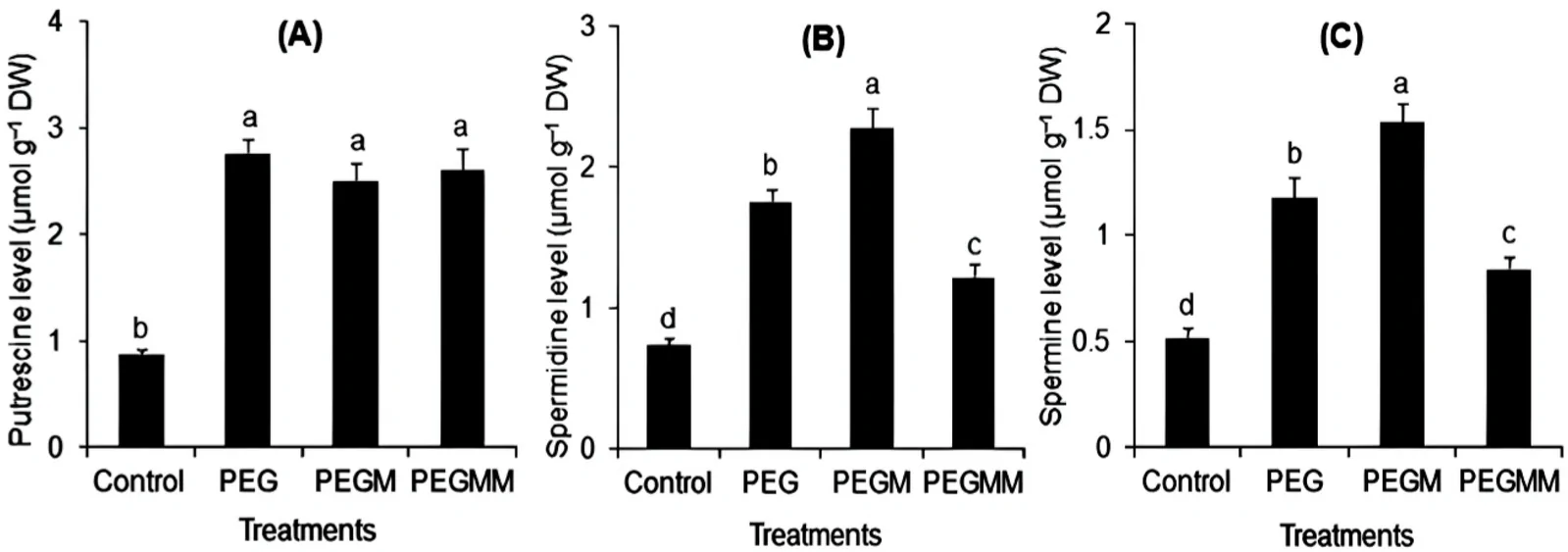
Changes in the contents of free polyamines ((**A**): putrescine; (**B**): spermidine; (**C**): spermine) in the leaves of barley seedlings treated with melatonin (DW: dry weight). Seedlings were cultured in full-strength (−0.15 MPa) Hoagland solution and marked as “Control”. Seedlings were cultured in full-strength Hoagland solution with PEG: polyethylene glycol (−0.85 MPa) (marked as “PEG”), PEG (−0.85 MPa) + melatonin (0.1 mM) (marked as “PEGM”), and PEG (−0.85 MPa) + melatonin (0.1 mM) + MGBG: methylglyoxyl-bis (guanylhydrazone) (0.5 mM) (marked as “PEGMM”), respectively. The data in the figure are the averages of nine independent tests ± standard error. Two-way analysis of variance (ANOVA) was used for significant-difference analysis among treatments, followed by Tukey’s Honest Significant Difference (HSD) test. The values associated the different lowercase letters over the column were significantly different according to HSD at *p* ≤ 0.05, while data of each column indicated by the same letters are not significantly different following Tukey’s HSD test (*p* > 0.05).

Three free polyamines, putrescine (Figure 3A), spermidine (Figure 3B), and spermine (Figure 3C), were assessed in this experiment in the barley leaves. Compared with the control, PEG brought about the increase in putrescine level, and PEG-induced increase in putrescine content was not affected by treatment with melatonin or melatonin plus MGBG (Figure 3A). However, the changes in spermidine and spermine contents were significant in the barley leaves under PEG, PEG plus melatonin, and PEG plus melatonin plus MGBG (Figure 3B,C). Compared with the control, PEG treatment increased the levels of both spermidine and spermine by about 0.5 times (statistical significance from “d” to “b”). Furthermore, melatonin treatment enhanced distinctly PEG-induced increases in the contents of spermine and spermidine (statistical significance from “b” to “a”). More interestingly, MGBG treatment inhibited the spermidine and spermine increases (statistical significance from “a” to “c”), which were induced by PEG and PEG plus melatonin (Figure 3B,C).

### 2.4. Change in the Proline Content in Barley Seedling Leaves Treated with Melatonin

**Figure 4:**
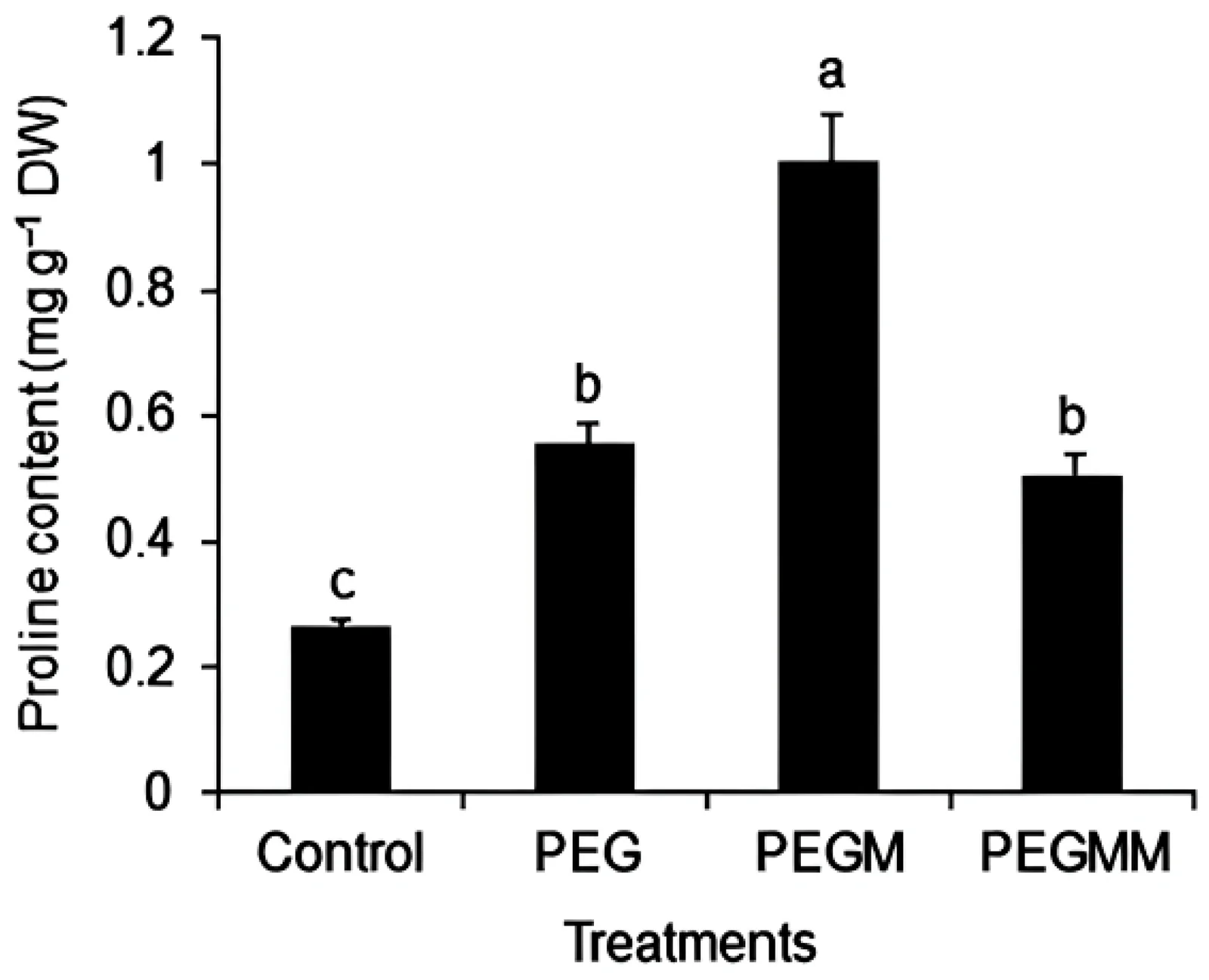
Change in the proline content in barley seedling leaves treated with melatonin (DW: dry weight). Seedlings were cultured in full-strength (−0.15 MPa) Hoagland solution and marked as “Control”. Seedlings were cultured in full-strength Hoagland solution with PEG (−0.85 MPa) (marked as “PEG”), PEG: polyethylene glycol (−0.85 MPa) + melatonin (0.1 mM) (marked as “PEGM”), and PEG (−0.85 MPa) + melatonin (0.1 mM) + MGBG (0.5 mM) (marked as “PEGMM”), respectively. The data in the figure are the averages of nine independent tests ± standard error. Two-way analysis of variance (ANOVA) was used for significant-difference analysis among treatments, followed by Tukey’s Honest Significant Difference (HSD) test. The values associated with the different lowercase letters over the column were significantly different according to HSD at *p* ≤ 0.05, while data of each column indicated by the same letters are not significantly different following Tukey’s HSD test (*p* > 0.05).

The osmotic adjustment substance, proline (Figure 4), was selected and detected in this study. As seen in Figure 4, compared with the control, PEG treatment elevated proline content from 0.26 to 0.55 mg g^−1^ DW in barley leaves, and melatonin enhanced the increase induced by PEG from 0.55 to 1.0 mg g^−1^ DW (statistical significance from “b” to “a”). However, MGBG inhibited melatonin-induced increase in proline content from 1.0 to 0.5 mg g^−1^ DW (statistical significance from “a” to “b”) (Figure 4). The results indicated that under PEG osmotic stress, exogenous melatonin could significantly increase the proline content in barley seedling leaves to enhance the osmotic regulation ability of barley seedlings.

### 2.5. Changes in the Activities of Superoxide Dismutase (SOD) and Ascorbate Peroxidase (APX) in Barley Leaves Treated with Melatonin

**Figure 5:**
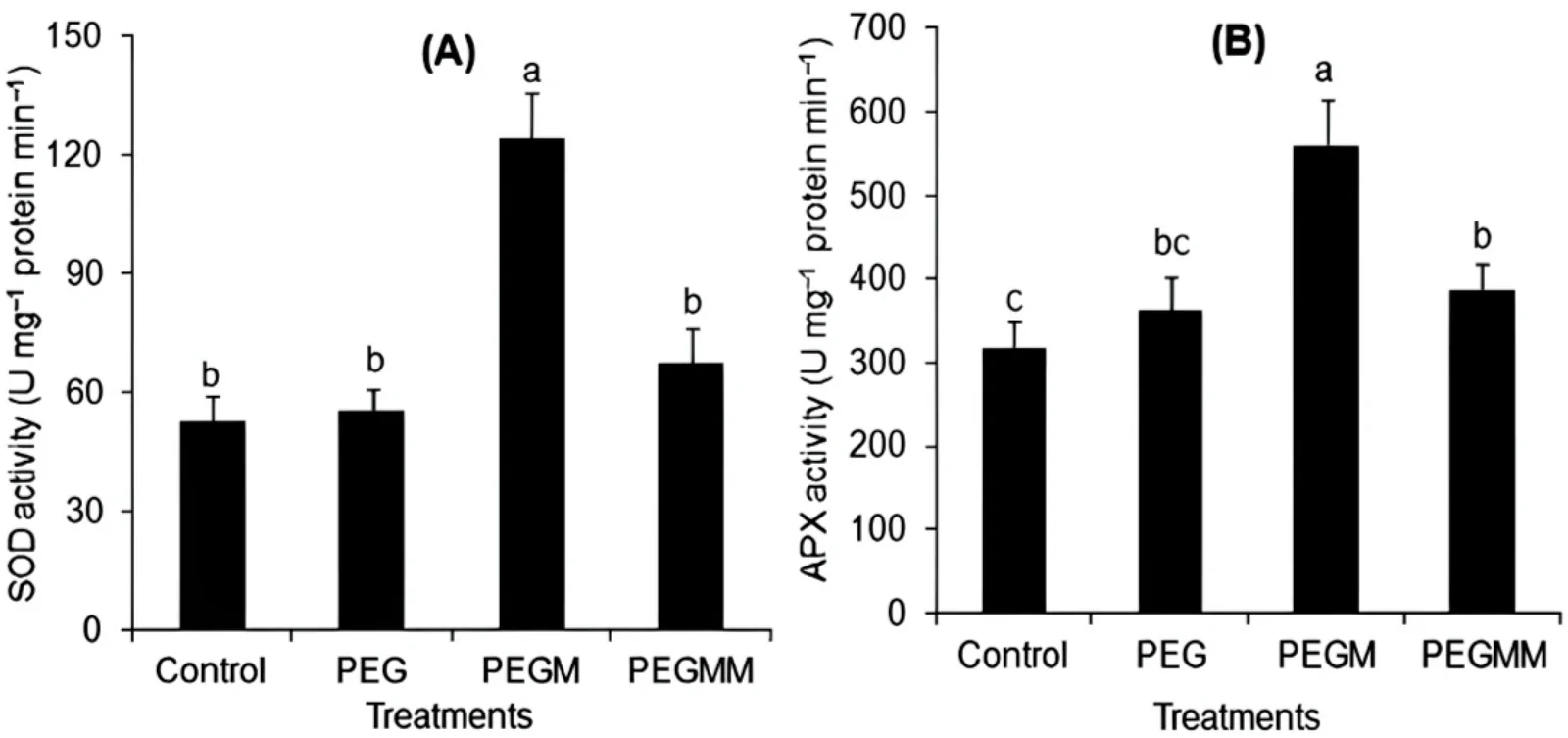
Changes in the activities of SOD (**A**) and APX (**B**) in barley leaves treated with melatonin. Seedlings were cultured in full-strength (−0.15 MPa) Hoagland solution and marked as “Control”. Seedlings were cultured in full-strength Hoagland solution with PEG: polyethylene glycol (−0.85 MPa) (marked as “PEG”), PEG (−0.85 MPa) + melatonin (0.1 mM) (marked as “PEGM”), and PEG (−0.85 MPa) + melatonin (0.1 mM) + MGBG: methylglyoxyl-bis (guanylhydrazone) (0.5 mM) (marked as “PEGMM”), respectively. The data in the figure are the averages of nine independent tests ± standard error. Two-way analysis of variance (ANOVA) was used for significant-difference analysis among treatments, followed by Tukey’s Honest Significant Difference (HSD) test. The values associated with the different lowercase letters over the column were significantly different according to HSD at *p* ≤ 0.05, while data of each column indicated by the same letters are not significantly different following Tukey’s HSD test (*p* > 0.05).

SOD and APX play important roles in eliminating reactive oxygen and maintaining chloroplast, so SOD and APX were selected and detected in this research. There was no significant difference in SOD activity between the control and PEG treatment. However, melatonin elevated SOD activity (statistical significance from “b” to “a”), and MGBG reversed the melatonin effect on SOD activity (statistical significance from “a” to “b”) (Figure 5A). APX shared the same trend of change in activity as that of SOD (Figure 5B) in barley seedling leaves under PEG, PEG plus melatonin, and PEG plus melatonin plus MGBG.

### 2.6. Effect of Melatonin on Oxidative Damage in Barley Leaves

**Figure 6:**
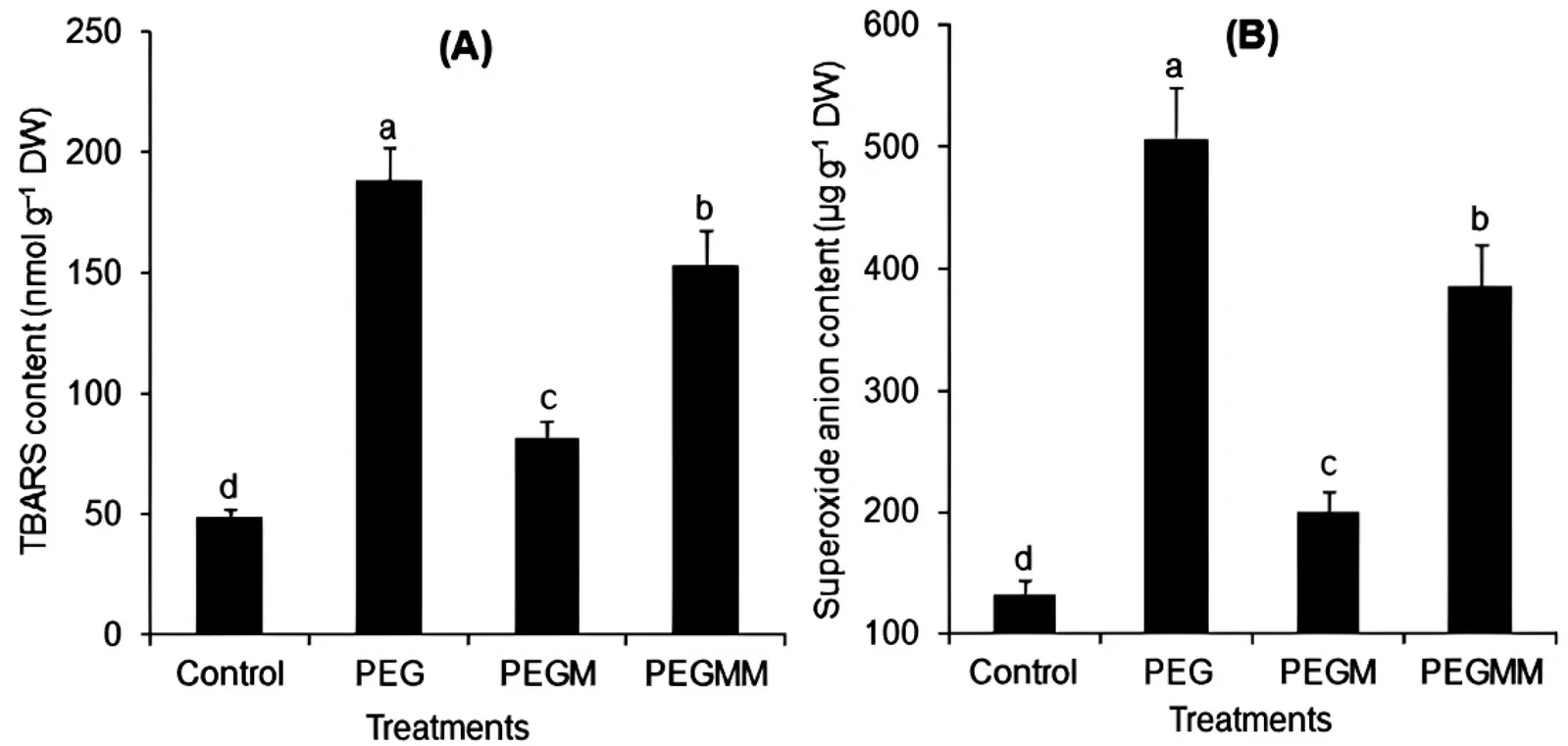
Effect of melatonin on oxidative damage in barley seedling leaves ((**A**): TBARS; (**B**): superoxide anion; DW: dry weight). Seedlings were cultured in full-strength (−0.15 MPa) Hoagland solution and marked as “Control”. Seedlings were cultured in full-strength Hoagland solution with PEG: polyethylene glycol (−0.85 MPa) (marked as “PEG”), PEG (−0.85 MPa) + melatonin (0.1 mM) (marked as “PEGM”), and PEG (−0.85 MPa) + melatonin (0.1 mM) + MGBG: methylglyoxyl-bis (guanylhydrazone) (0.5 mM) (marked as “PEGMM”), respectively. The data in the figure are the averages of nine independent tests ± standard error. Two-way analysis of variance (ANOVA) was used for significant-difference analysis among treatments, followed by Tukey’s Honest Significant Difference (HSD) test. The values associated with the different lowercase letters over the column were significantly different according to HSD at *p* ≤ 0.05, while data of each column indicated by the same letters are not significantly different following Tukey’s HSD test (*p* > 0.05).

Two typical parameters, thiobarbituric acid reactive substance (TBARS, Figure 6A) and superoxide anion (Figure 6B), which could show the extent of oxidative damage, were selected and detected in this study. Compared with the control, PEG treatment obviously elevated the levels of TBARS and superoxide anion. Treatment with melatonin restrained the PEG-osmotic-stress-induced increases in both TBARS and superoxide anion (statistical significance from “a” to “c”). However, MGBG treatment reversed melatonin effects on the contents of TBARS and superoxide anion (statistical significance from “c” to “b”) (Figure 6). The results indicated that PEG brought about oxidative damage in barley leaves and that exogenous melatonin could alleviate the damage. The effect of melatonin was counteracted by MGBG. These results, as shown in Figure 5 and Figure 6, suggested that exogenous melatonin could alleviate PEG-induced oxidative damage in barley leaves by elevating the activities of SOD and APX. The increase in antioxidant ability might result in improving photosynthetic capacity, so the photosynthetic performances of barley seedling leaves treated with melatonin were also elucidated as follows.

### 2.7. Changes in Photosynthetic Performances of Barley Seedling Leaves Treated with Melatonin

**Figure 7:**
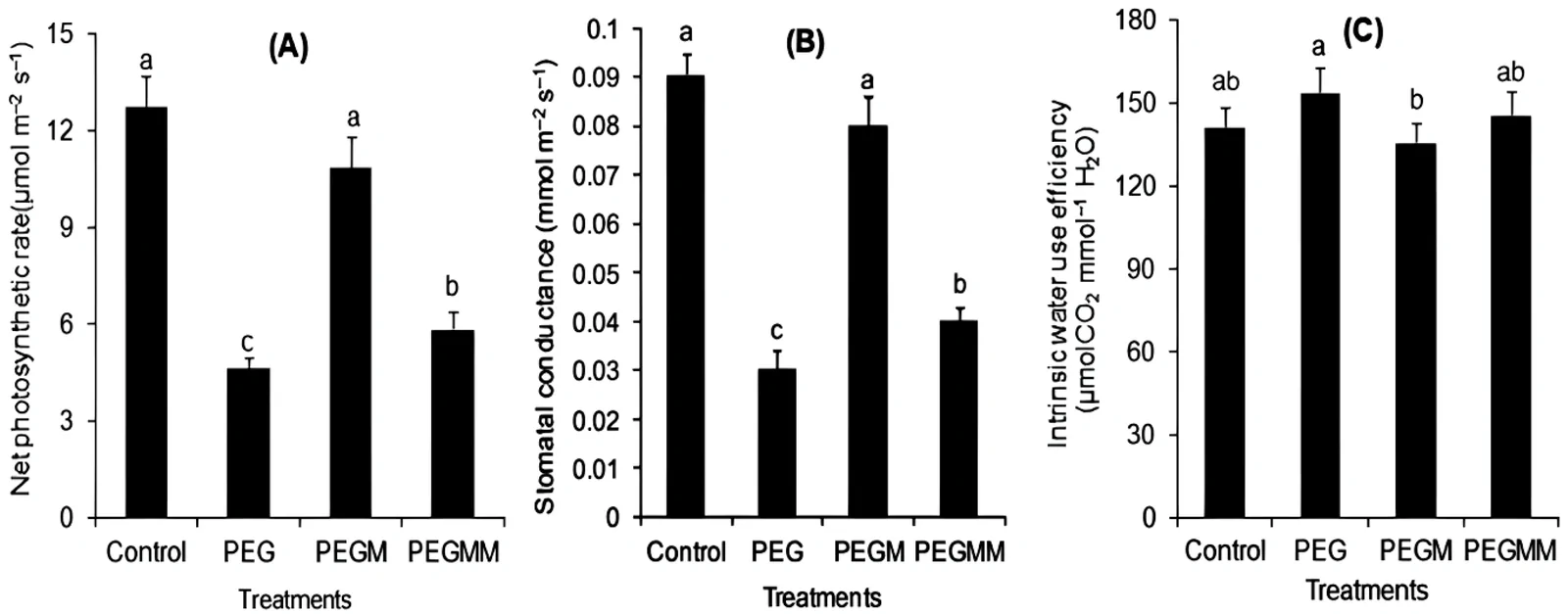
Changes in photosynthetic performances ((**A**): net photosynthetic rate; (**B**): stomatal conductance; (**C**): intrinsic water-use efficiency) of barley seedling leaves treated with melatonin. Seedlings were cultured in full-strength (−0.15 MPa) Hoagland solution and marked as “Control”. Seedlings were cultured in full-strength Hoagland solution with PEG: polyethylene glycol (−0.85 MPa) (marked as “PEG”), PEG (−0.85 MPa) + melatonin (0.1 mM) (marked as “PEGM”), and PEG (−0.85 MPa) + melatonin (0.1 mM) + MGBG: methylglyoxyl-bis (guanylhydrazone) (0.5 mM) (marked as “PEGMM”), respectively. The data in the figure are the averages of nine independent tests ± standard error. Two-way analysis of variance (ANOVA) was used for significant-difference analysis among treatments, followed by Tukey’s Honest Significant Difference (HSD) test. The values associated with the different lowercase letters over the column were significantly different according to HSD at *p* ≤ 0.05, while data of each column indicated by the same letters are not significantly different following Tukey’s HSD test (*p* > 0.05).

Although many parameters are involved in photosynthetic performance, such as chlorophyll content, intercellular CO_2_ concentration, transpiration rate, chlorophyll fluorescence, and effective quantum yield, net photosynthetic rate and stomatal conductance are more representative and widely used to assess the photosynthetic performance of plant leaves. Therefore, in this study, they were selected to assess the photosynthetic performance of barley seedlings. PEG treatment reduced net photosynthetic rate (statistical significance from “a” to “c”) (Figure 7A) and stomatal conductance (statistical significance from “a” to “c”) (Figure 7B). The PEG-osmotic-stress-induced reduction was mitigated by exogenous melatonin application (statistical significance from “c” to “a”), and the melatonin effect was reversed by inhibitor MGBG (statistical significance from “a” to “b”) (Figure 7). These results showed that exogenous melatonin could improve the photosynthetic performance of barley leaves. In addition, from the change in intrinsic water-use efficiency, it was observed that melatonin could alleviate the adverse impact of PEG osmotic stress on barley seedlings (Figure 7C).

## 3. Discussion

### 3.1. Melatonin and Tolerance

It has been well reported that melatonin can improve plant tolerance against environmental stresses, especially against drought stress [9,10,11,12,13]. In the present research, PEG (−0.85 MPa) osmotic stress, which was used to simulate drought stress, reduced the growth of barley seedlings, as judged by the two typical parameters, root length (Figure 1A) and RIRBSDW (Figure 1B). From Figure 1, it can be clearly seen that melatonin could alleviate the inhibition effect of PEG on the growth of barley seedlings, and thereby improve the tolerance of barley seedlings against osmotic stress, which was further reinforced by the change in relative water content (Supplementary Data S3). Our finding is well consistent with those of Dzinyela et al. [10], Zhang et al. [11], Hu et al. [12], and Noor and Sheng [13].

In those studies, the mechanism of melatonin-mediated drought tolerance has been well documented. Noor and Sheng [13] have reported that exogenous melatonin markedly enhanced maize photosynthetic characteristics by up-regulating CO_2_ assimilation genes and related enzymes. Concurrently, the activities of antioxidant enzymes and the Halliwell–Asada cycle are activated by melatonin, leading to marked decreases in the levels of H_2_O_2_ and TBARS. The research of Hu et al. [12] has also showed that 0.1 mM melatonin alleviates drought stress by improving photosynthetic performance and antioxidant capacity in *Leymus chinensis*. Besides by improving antioxidant capacity and photosynthetic performance, melatonin It does the same for licorice (*Glycyrrhiza uralensis* Fisch.) under PEG osmotic stress, as well as elevating seedling tolerance by up-regulating aquaporin genes, as described by Zhang et al. [11]. Although melatonin-mediated phytohormonal crosstalks are involved in improving plant tolerance against drought stress [10], polyamine metabolism is scarcely mentioned in studies on the mechanism of melatonin-mediated drought tolerance. In the present research, we found that exogenous melatonin mitigated osmotic stress by elevating the proline level (Figure 4) and the activities of antioxidant enzymes (Figure 6), and thereby alleviating oxidative damage (Figure 5) and improving photosynthetic performance (Figure 7) in barley seedling leaves, which was consistent with the previous studies. Furthermore, we found that polyamine metabolism was involved in melatonin-mediated osmotic stress tolerance and that SAMDC played pivotal functions in barley seedlings.

### 3.2. Melatonin-Induced Polyamine Metabolism and Osmotic Adjustment Substances

Osmotic regulation is an important response mechanism of plants to abiotic stresses. Under drought stress, plants can accumulate various organic or inorganic substances, such as amino acids, quaternary amines, sugars, and sugar alcohols, which are important osmotic regulatory substances, to enhance water absorption, maintain the stability of the internal environment of cells, and protect normal metabolic processes within the plants [32,33]. In this study, the osmotic adjustment substance, proline, increased in the barley seedling leaves under PEG osmotic stress, and melatonin enhanced the PEG-induced increase (Figure 4). Osmotic adjustment has been established as a physiological adaptation strategy alongside drought tolerance [34]. Sharma et al. [35] have reported that barley seedlings with high osmotic adjustment capacities can preserve cell turgor pressure and plant water status alongside higher productivity, while those with low osmotic adjustment capacity have lower yields. This encompasses the hyper-accumulation of osmotic regulatory substances, such as proline and so on. It is well documented that osmotic regulatory substances are highly soluble molecules, free of net charge within the physiological pH range, non-toxic at molar concentrations, and involved in several metabolic pathways related to enhancing water influx and maintaining cell turgor pressure and osmotic adjustment capacity [36]. Therefore, in the barley seedling leaves, PEG osmotic stress induced the accumulation of proline to enhance water influx and seedling tolerance to osmotic stress.

More importantly, we found that melatonin promoted PEG-induced increases in SAMDC activity (Figure 2B) and the contents of spermine (Figure 3B) and spermidine (Figure 3C), while the PEG-induced increase in arginine decarboxylase activity (Figure 2A) and putrescine level (Figure 3A) were not affected by melatonin treatment. These results hint that melatonin functions by elevating the activity of SAMDC and thereby the contents of spermine and spermidine. The notion is reinforced by the experimental result with MGBG inhibitor. MGBG inhibited the PEG- and melatonin-induced increases not only in SAMDC activity (Figure 2A) and the contents of spermidine and spermine (Figure 3B,C), but also in the osmotic regulatory substance, proline (Figure 4), and thereby the tolerance of barley seedlings to osmotic stress (Figure 1). Although MGBG may exert pleiotropic effects and there may be potential off-target effects, the present results of MGBG application might supply indirect evidence for our notion. There was no change in putrescine level in the barley seedlings with melatonin treatment. It was supposed that with the melatonin-induced increase in SAMDC activity, putrescine might be converted into spermine and spermidine. Thus, putrescine did not show the same response as spermidine and spermine under PEG, PEG plus melatonin, and PEG plus melatonin plus MGBG. Our previous study [37] has also shown that the conversion from putrescine to spermine and spermidine plays important functions in wheat plant tolerance against soil drought stress. Our study is strongly consistent with the previous works [29].

### 3.3. Melatonin-Induced Polyamine Metabolism and Antioxidant Ability, as Well as Photosynthetic Performance

Under drought stress, an imbalance between the generation and removal of reactive oxygen species in plant cells has been shown to result in damage to the membrane system. Drought stress often leads to a decline in the antioxidant capacity and photosynthetic ability of plants. The application of antioxidants can maintain the balance of reactive oxygen species in cells. As a natural antioxidant, melatonin can increase the content and activity of antioxidant substances in plants, thereby alleviating the effects of drought stress on them. Likewise, in this study, osmotic stress led to increases in TBARS (Figure 6A) and superoxide anion (Figure 6B), indicating that barley seedlings were subjected to oxidative damage, as TBARS is an important parameter showing lipid peroxidation of the bio-membranes. Concurrently, photosynthetic ability of barley seedling leaves decreased under PEG stress (Figure 7). Melatonin treatment markedly elevated the activities of SOD (Figure 5A) and APX (Figure 5B), decreased the levels of TBARS (Figure 6A) and superoxide anion (Figure 6B), and thereby elevated net photosynthetic rate (Figure 7A) and stomatal conductance (Figure 7B), showing that melatonin alleviated oxidative damage (Figure 5), elevated antioxidant capacity (Figure 6), and thereby improved the photosynthetic performance of barley seedlings (Figure 7).

To further probe the mechanism of melatonin-mediated osmotic stress resistance, polyamine metabolism was elucidated in this study. Melatonin markedly elevated the activity of SAMDC (Figure 2B) and thereby elevated spermidine (Figure 3B) and spermine (Figure 3C) contents in barley seedling leaves. It is speculated that melatonin functions by elevating SAMDC activity in barley seedlings. The hypothesis is reinforced by the additional experimental results with MGBG inhibitor. MGBG treatment not only decreased SAMDC activity (Figure 2B), coupled with the decreases in the levels of spermidine (Figure 3B) and spermine (Figure 3C), but also reduced the antioxidant capacity (Figure 5 and Figure 6) and photosynthetic performance (Figure 7) of barley seedlings. Our previous study [14] has also shown that melatonin application can improve the resistance of peach fruit to cold stress by elevating the activity of SAMDC and thereby the contents of spermine and spermidine. The present result is also consistent with the previous studies, which show that an exogenous application of melatonin can mitigate reactive-oxygen-species-induced oxidative injury by elevating SOD and APX activities in soybean (*Glycine max*) plants under drought stress, which can provide a better cellular reduction–oxidation state for plant growth [38].

The reason why spermidine and spermine factor into melatonin-mediated tolerance might be attributed to the elimination of reactive oxygen species. First, spermidine and spermine can eliminate directly reactive oxygen species. Drolet et al. [39] demonstrated that superoxide anions can be significantly inhibited by spermidine and spermine. Moreover, the production of superoxide anion by senescent microsomal membranes can also be inhibited by spermidine and spermine. Furthermore, the clearance efficiency of superoxide anion is determined by the degree of amination of polyamines. Drolet et al. also argue that the physiological functions of polyamine, such as inhibiting membrane lipid peroxidation and delaying aging, are attributed to its direct scavenging ability of reactive oxygen species. Additionally, the study of Khan et al. [40] suggested that polyamines can directly eliminate reactive oxygen species. Second, spermidine and spermine can scavenge indirectly reactive oxygen species by influencing other intermediate substances, especially the enzymatic and non-enzymatic systems that eliminate reactive oxygen species, thereby enhancing the stress resistance of plants and delaying aging. The mechanism by which polyamines enhance the activity of antioxidant enzymes in plants can be visualized as follows. On the one hand, polyamines can induce the biosynthesis of antioxidant enzymes. On the other hand, polyamine can directly act on enzyme molecules to enhance enzyme activity. Third, polyamines can reduce the rate of reactive oxygen species production. Tadolini [41] has reported that polyamines can be bound to bio-membranes to form ternary complexes with membrane phospholipids and Fe^2+^ and thereby reduce the Fe^2+^-mediated production of reactive oxygen species. Shen et al. [42] found that the spermidine in the leaves of cucumber seedlings can inhibit the increase in reactive oxygen species levels by suppressing the increase in NADPH oxidase activity induced by cold stress and thereby enhance the cold stress resistance of the seedlings. In short, the mechanism by which spermidine and spermine eliminate reactive oxygen species has been explored in the previous literature [41,42].

## 4. Materials and Methods

### 4.1. Barley Cultivar Selection, Seedling Cultivation, and Osmotic Stress Treatments

“Su Beer” No. 6 barley (*Hordeum vulgare* L.) cultivar was used in this experiment. The cultivar is bred by the Agricultural Science Academy, Nanjing, China. Although the barley of this cultivar is high-yield, it is a drought-sensitive cultivar. Thus, it is important to improve its drought resistance. The results of our preliminary experiments indicated that the appropriate concentration of melatonin (0.1 mM) application could elevate the osmotic stress tolerance (Supplementary Data S1). The barley seeds were sterilized with HgCl_2_ (0.2%, *w*/*v*) for 15 min, rinsed with de-ionized water, and germinated in on three layers of wet filter paper for 3 d. Then, the selected 20 seeds were uniformly sowed in black plastic cups (height/rim diameter/bottom diameter: 20 cm/15 cm/10 cm; volume: 2487 cm^3^) with quartz sand and small holes in the bottoms (10 seeds/cup). These plastic cups were then placed in large black turnover boxes with half-strength Hoagland solution in a controlled-environment chamber with a 25/15 °C (day/night) temperature period, a 14 h photoperiod of 400 μmol m^−2^ s^−1^, and 60% relative humidity. The half-strength Hoagland solution was renewed every day. When the third leaf was fully expanded, the seedlings were cultivated with full-strength Hoagland solution and treated with the reagents, PEG, melatonin, and MGBG. The appropriate concentration of MGBG was determined by our preliminary experiments (Supplementary Data S2).

Control: Seedlings were cultured in the full-strength Hoagland solution (−0.15 MPa) and marked as “Control”.

PEG treatment: Seedlings were cultured in full-strength Hoagland solution with PEG (−0.85 MPa), which was marked as “PEG” in the figures.

PEG + melatonin treatment: Seedlings were cultured in full-strength Hoagland solution with PEG (−0.85 MPa) and melatonin (0.1 mM), which was marked as “PEGM” in the figures.

PEG + melatonin + MGBG treatment: Seedlings were cultured in full-strength Hoagland solution with PEG (−0.85 MPa), melatonin (0.1 mM), and MGBG (0.5 mM), which was marked as “PEGMM” in the figures.

PEG, melatonin, and MGBG were all bought from Sigma Chemical Co. The water potentials of the full-strength Hoagland solution of the “Control” and “Treatments” were determined with an Osmometer (Beijing Zhonghui Tiancheng Technology Co., Ltd, Beijing, China, Model: TEN60). Water potentials of −0.15 and −0.85 MPa, the timing, sequence, and doses of PEG, melatonin, and MGBG application, and the number of barley seedlings constituting each experimental unit were established according to our preliminary experiments. After the seedlings were treated for 7 d, the seedlings in three randomly selected cups with the same treatment were sampled and tested immediately.

### 4.2. Evaluation of Root Length and RIRBSDW

The root length of the barley seedling was detected by a ruler, and centimeter (cm) was used as the unit. RIRBSDW was assessed by the method of Du et al. [43]. The increasing rate of barley seedling dry weight (IRBSDW) = (DW_7_ − DW_0_)/DW_0_ (DW_7_ and DW_0_ indicated the dry weight of barley seedlings treated with the reagents for 7 d and 0 d, respectively). Then, RIRBSDW was assessed by the following equation:

RIRBSDW (%) = IRBSDW of every treatment/IRBSDW of the control × 100.

### 4.3. Assessment of the Activities of Arginine Decarboxylase and SAMDC

The activity of arginine decarboxylase was examined by the method of Du et al. [37] with minor modifications: Homogenize seedling leaves (1 g) in cool extract solution, including phosphate buffer (pH 6.5, 50 mM), ethylene diamine tetra-acetic acid (5 mM), vitamin E (20 mM), phenyl methyl sulfonyl fluoride (0.1 μM), phosphopyridoxal (40 μM), and dithiothreitol (5 mM). Precipitate the solution in (NH_4_)_2_SO_4_ (20–50%), re-dissolve the precipitate in phosphate buffer (pH 6.5, 10 mM), dialyze it for 12 h, and centrifuge the sample at 25,000× *g* for 15 min. Arginine decarboxylase was in the supernatant. The parameter was expressed on a dry weight (DW) basis to avoid the influence of water content on the observed differences. Thus, enzyme activity unit was shown as nmol g^−1^ dry weight (DW) h^−1^. Similarly, the following SAMDC activity and the levels of polyamines, proline, TBARS, and superoxide ion were also expressed on a DW basis.

SAMDC activity was detected by the method of Du et al. [44] with some modifications and was assessed according to the released ^14^CO_2_ level. The assessing mixture contained Tris-HCl buffer (100 mM, pH 8.3), 2-mercaptoethanol (1.0 mM), 0.1 mM EDTA (0.1 mM), and the prepared enzyme solution. In a glass scintillation vial, the reaction was carried out to trap the released ^14^CO_2_. After the reaction for 5 min at 30 °C, S-adenosyl-L-[carboxyl-^14^C] methionine (0.5 nmol) was added to the assessing mixture, and the vial was capped immediately. After the reaction for 30 min, it was stopped by adding 0.3 mL KH_2_PO_4_ (1 M) into the mixture. Then the vial was immediately closed again and shaken for 1 h at 37 °C to enhance the release of CO_2_. The released ^14^CO_2_ was collected and transferred to another vial with 5 mL toluene-based scintillation fluid and determined. The enzyme activity unit was shown as μmol ^14^CO_2_ g^−1^ DW s^−1^.

### 4.4. Analysis of the Polyamines in the Leaves of Barley Seedlings

The contents of free putrescine, spermine, and spermidine were measured by the method of Du et al. [44] with minor modifications. The barley seedling leaves (1 g) were homogenized in 5% (*v*/*v*) ice-cold HClO_4_. The homogenate was then incubated for 2 h at 4 °C and centrifuged for 30 min at 13,000× *g*. The free polyamines were included in the supernatant. They were benzoylated using benzoyl chloride. We extracted the benzoylated polyamines above in cold diethyl ether and centrifuged at 2500× *g* for 15 min. We air-dried the benzoylated polyamines and re-dissolved them in 1 mL methanol (64%, *v*/*v*). The free polyamines were detected by HPLC (Waters 2695, Waters Co., Miford, MA, USA). The unit of polyamine content was shown as μmol g^−1^ DW.

### 4.5. Determination of Proline

The proline content in the barley seedling leaves was determined by the sulfosalicylic acid method [45], and mg g^−1^ DW was used as one unit. The detected result was compared to the standard constructed curve using a known amount of proline.

### 4.6. Assessment of SOD and APX

The activity of SOD was assessed by the method of Giannopolitis and Ries [46]. By noting the photochemical reduction rate of Nitro-Blue tetrazolium chloride at 560 nm, SOD activity was determined. The assay mixture contained phosphate buffer (25 mM, pH 6.8), Nitro-Blue tetrazolium chloride (75 μM), L-methionine (12 mM), Na-carbonate (50 mM, pH 10.2), riboflavin (1 μM), and enzyme extract in a total volume of 3 mL. The blank mixture had the same composition but was kept in the dark. The activity of APX was assessed spectrophotometrically at 290 nm by the method of Nakano and Asada [47]. The reduced forms of ascorbic acid and glutathione were determined by using the kit (Art. No. ASA-1-W and Art. No. GSH-1-W) based on the method of 5,5-dithiobis-(2-nitrobenzoic acid) and the red phenanthroline method in accordance with the manufacturer’s instructions (Suzhou Comin Biotechnology Co., Ltd., Suzhou, China), respectively. U mg^−1^ protein min^−1^ was used as one unit of SOD or APX activity. The protein content was determined by the Coomassie brilliant blue G-250 staining method [48], and the detected result was compared to the standard constructed curve using a known amount of protein.

### 4.7. Determination of TBARS and Superoxide Anion Contents

TBARS content was determined with thiobarbituric acid by the method of Alexieva et al. [49], and nmol g^−1^ DW was used as one unit. Superoxide anion content was assessed with hydroxylamine by the method of Lukatkin [50] and was noted spectrophotometrically at 530 nm. One unit of superoxide anion was defined as μg g^−1^ DW.

### 4.8. Analysis of Leaf Photosynthetic Characteristics

The third fully-expanded 9 seedling leaves were randomly selected as samples to assess leaf photosynthetic characteristics. A portable photosynthetic system (Model: LI-6800, LI-COR Inc., Lincoln, NE, USA) was used to detect net photosynthetic rate and stomatal conductance between 9:00 AM and 10:00 AM with a light source of 1500 μmol/(m^2^·s), relative humidity of 70%, leaf chamber at 30 °C, air velocity of 500 μmol/s, and CO_2_ concentration of 400 μmol/mol; μmol m^−2^ s^−1^ and mmol m^−2^ s^−1^ were used as units of photosynthetic rate and stomatal conductance, respectively.

### 4.9. Statistical Analysis

The experiment was conducted three times, i.e., three biological replicates and three technical replicates were carried out for every biological replicate. Thus, the data in each figure are the averages of nine independent tests ± standard error. Data analysis was performed using Microsoft Excel and SPSS 20.0 software (SPSS Inc., Armonk, NY, USA). Two-way analysis of variance was used for significant-difference analysis among treatments, followed by Tukey’s Honest Significant Difference (HSD) test. The values associated with the different lowercase letters over the column were significantly different according to HSD at *p* ≤ 0.05, while data of each column indicated by the same letters over the column are not significantly different following Tukey’s HSD test (*p* > 0.05).

## 5. Conclusions

**Figure 8:**
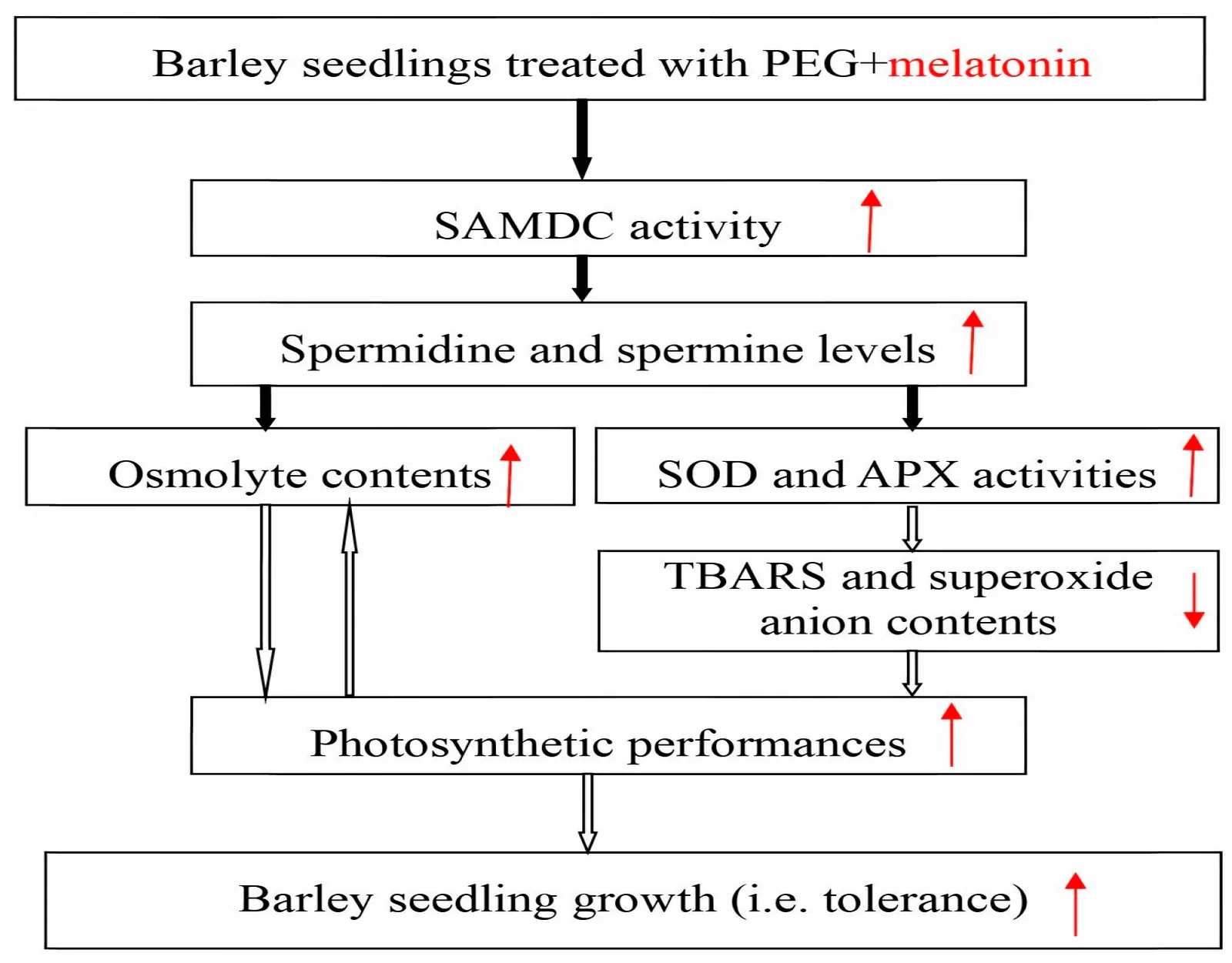
Thumbnail sketch of the study. The black solid arrow presents the causal relationship explored in this study. The hollow arrow presents the causal relationship explored by previous studies. A red upward arrow indicates that a trend is increasing. A red downward arrow indicates that a trend is decreasing.

Melatonin-mediated resistance of barley seedlings to osmotic stress is further unveiled in this study. Melatonin application elevates SAMDC activity in barley leaves, which leads to increases in the contents of spermine and spermidine. With the increasing spermidine and spermine, the osmotic adjustment and antioxidant capacity increase, and thereby the photosynthetic performance is improved. In brief, SAMDC might be involved in the melatonin-mediated tolerance of barley seedlings to osmotic stress. To illuminate the study more clearly, a thumbnail diagram is presented in Figure 8. A preliminary theoretical study has been conducted in this paper. However, in the interest of practical application, as an agrochemical for improving crop drought tolerance in water-limited environments, melatonin of an optimum concentration should be explored for different crops at different growth stages. It is undoubted that the mechanism of melatonin-mediated plant tolerance against drought stress is rather complicated and that phytohormone crosstalk, gene expression, and the adjustments of related metabolisms are involved in the process. In the future, with the accumulation of more and more evidence on the subject, a fuller picture will be unfolded before our eyes.

## Data Availability

The original contributions presented in this study are included in the article. Further inquiries can be directed to the corresponding author.

## Supplementary Materials

The following supporting information can be downloaded at: https://www.mdpi.com/article/10.3390/plants15203114/s1. Supplementary Data S1 (S1: The selection of 0.1 mM melatonin in the preliminary experiment); Supplementary Data S2 (S2: The selection of 0.5 mM MGBG in the preliminary experiment); Supplementary Data S3 (S3: The changes in the relative water content of the barley seedling leaves in the preliminary experiment).

## Abbreviations

APX	Ascorbate peroxidase
DW	Dry weight
HPLC	High-pressure liquid chromatography
MGBG	Methylglyoxyl-bis (guanylhydrazone)
PEG	Polyethylene glycol
RIRBSDW	Relative increasing rate of barley seedling dry weight
SAMDC	*S*-adenosylmethionine decarboxylase
SOD	Superoxide dismutase
TBARS	Thiobarbituric acid reactive substance
