# Supplementary material for: SAMDC Activity Is Involved in the Melatonin-Mediated Tolerance of Barley (Hordeum vulgare L.) Seedlings to Osmotic Stress
Source: Plants (Basel). 2026 Oct 12;15(20):3114. doi: 10.3390/plants15203114 (PMC13708447; doi:10.3390/plants15203114)
Supplement: Supplementary file 1 [file plants-15-03114-s001.zip › Supplementary data S1.pdf]

plants

MDPI

Supplementary data S1 (The selection of 0.1 mM melatonin in the preliminary experiment)

Table S1. Changes in root length and relative increasing rate of barley seedling dry weight (RIRBSDW) of seedlings under PEG osmotic stress (-0.85 MPa) plus melatonin at different concentration.

Seedling growth	Melatonin concentrations (mM)					
	0	0.01	0.05	0.1	0.15	0.20
Root length (cm)	9.3±0.7 c	10.4±0.8 bc	11.2±0.9 b	16.9±1.4 a	18.6±2.3 a	17.5±2.1 a
RIRBSDW(%)	41±3.9c	43±4.2 bc	45±4.7 b	83±9.1 a	86±9.4 a	84±8.2 a

Note: The data in the table are the averages of nine independent tests ± standard error. Tukey test was applied to compare significant differences among the means at the level of $P < 0.05$ and the lowercase letters indicated the significant differences, and the lowercase letters indicated the significant differences. After the seedlings were treated for 7 d, the seedlings in three selected randomly cups with the same treatment were sampled and tested immediately. From the table, it could be observed that 0.1 mM of melatonin enhanced markedly ($P < 0.05$) the growth of barley seedlings under osmotic stress. Therefore, 0.1 mM of melatonin was selected to use in the study.
