# Supplementary material for: SAMDC Activity Is Involved in the Melatonin-Mediated Tolerance of Barley (Hordeum vulgare L.) Seedlings to Osmotic Stress
Source: Plants (Basel). 2026 Oct 12;15(20):3114. doi: 10.3390/plants15203114 (PMC13708447; doi:10.3390/plants15203114)
Supplement: Supplementary file 1 [file plants-15-03114-s001.zip › Supplementary data S2.pdf]

Supplementary data S2 (The selection of 0.5 mM MGBG in the preliminary experiment)

Table S2. Change in SAMDC activity in the leaves of barley seedling under PEG (-0.85 MPa) + melatonin (0.1 mM) + MGBG at different concentration.

	MGBG concentration (mM)					
	0	0.1	0.3	0.5	1.0	1.5
SAMDC activity (μmol $^{14}CO_2$ g^{-1} dry weight s^{-1})	41.6 ±3.5 a	33.2±2.9 b	27.6±2.2 c	16.7±1.5 d	16.1±1.3 d	15.8±1.0 d

Note: The data in the table are the averages of nine independent tests ± standard error. Tukey test was applied to compare significant differences among the means at the level of $P < 0.05$ and the lowercase letters indicated the significant differences. When the third leaf was fully expanded, barley seedlings were cultured in the Hoagland solution of full-strength with PEG (-0.85 MPa) + melatonin (0.1 mM) + MGBG (at different concentration as mentioned in the table) for 7 d. Then, the third leaf (1 g) was sampled, and SAMDC activity in the leaf was assessed. From the table, it could be observed that was inhibited by MGBG, and with the increase in MGBG concentration, the inhibition effect increased. There was no significant difference in the inhibition effect at 0.5, 1.0 and 1.5 mM MGBG concentration. Therefore, 0.5 mM MGBG was selected to use in our study.
