# Supplementary material for: SAMDC Activity Is Involved in the Melatonin-Mediated Tolerance of Barley (Hordeum vulgare L.) Seedlings to Osmotic Stress
Source: Plants (Basel). 2026 Oct 12;15(20):3114. doi: 10.3390/plants15203114 (PMC13708447; doi:10.3390/plants15203114)
Supplement: Supplementary file 1 [file plants-15-03114-s001.zip › Supplementary data S3.pdf]

plants

MDPI

Supplementary data S3

Table S3. The changes in the relative water content (RWC) of the barley seedling leaves in the preliminary experiment.

	Treatments			
	**Control**	**PEG**	**PEGM**	**PEGMM**
RWC (%)	96±8.7 a	71±6.4 c	89±7.2 b	73±6.3 c

Note: The data in the table are the averages of nine independent tests ± standard error. Tukey test was applied to compare significant differences among the means at the level of $P < 0.05$ and the lowercase letters indicated the significant differences, and the lowercase letters indicated the significant differences. RWC was assessed by the following formula: RWC (%) = (fresh weight – dry weight) / (saturation weight – dry weight) × 100 (In the formula, fresh weight, saturation weight and dry weight represent the weight of the seedling leaves treated for 7 d, respectively). After being weighed for fresh weight, the fresh leaves were immersed in distilled water immediately for fully absorbing water until the leaf weight was constant. Then, the weight was defined as saturation weight. The samples of the fresh leaves were put into an oven, killed out at 110 °C for 20 minutes, and then baked at 65 °C until the leaf weight was constant. The constant leaf weight was regarded as dry weight. Seedlings were cultured in the Hoagland solution of full-strength (-0.15 MPa) and marked as "Control". Seedlings were cultured in the Hoagland solution of full-strength with PEG (-0.85 MPa) (marked as "PEG"), PEG (-0.85 MPa) + melatonin (0.1 mM) (marked as "PEGM"), and PEG (-0.85 MPa) + melatonin (0.1 mM) + MGBG (0.5 mM) (marked as "PEGMM"), respectively.
